# Der Bayerische Landtag und die Homöopathie – ein kritischer Kommentar zum Antrag „Todesfälle durch multiresistente Keime vermeiden IV“ (Drucksache 18/3320)

**DOI:** 10.1007/s00481-020-00593-z

**Published:** 2020-07-20

**Authors:** Yannick Borkens, Yannik Plasberg

**Affiliations:** 1 College of Public Health, Medical and Veterinary Science, James Cook University, 1 James Cook Drive, 4811 Townsville, Queensland Australien; 2 Düsseldorf, Nordrhein-Westfalen Deutschland

Anfang August 2019 reichten Abgeordnete der Christlich-Sozialen Union (CSU) und der Freien Wähler den Antrag „Todesfälle durch multiresistente Keime vermeiden IV – Studie zu einem reduzierten Antibiotikaeinsatz“ im Bayerischen Landtag ein. Der Antrag diskutiert, wie alternativmedizinische und homöopathische Präparate den Einsatz von Antibiotika reduzieren oder verhindern könnten. Die Antragsteller fordern unter anderem eine Studie, die diese Frage erforschen soll (Seidenath et al. 2019a). Diesem Antrag und somit auch der Studie wurde am 07.11.2019 zugestimmt (Ärzteblatt 2019). Dieser Vorgang wirft ethische Probleme auf, die wir in diesem Kommentar erläutern und diskutieren möchten.

## Hintergrund

Krankenhauskeime und antibiotikaresistente Bakterien entwickeln sich zu einem ernsten medizinischen Problem. Die Weltgesundheitsorganisation (WHO) schätzt, dass die Wahrscheinlichkeit, an einer Infektion mit resistenten Pathogenen zu sterben, 64 % höher ist als an einer Infektion mit nichtresistenten Pathogenen (WHO 2018). In Berlin starben in den letzten Jahren 534 Menschen an Krankenhauskeimen (Ärzteblatt 2018). Durch die steigende Nutzung von Antibiotika haben Bakterien und andere Pathogene die Möglichkeit, sich an diese anzupassen (Dodds 2017).

Um das Problem zu lösen und die Gefahr für Patienten zu minimieren, sind nicht nur schnelle Maßnahmen, sondern auch komplett neue Therapieansätze und Strategien erforderlich (Bragg et al. 2018; van Puyvelde et al. 2018). Zu diesen Ansätzen gehört etwa die Nutzung von UV-Licht (Kinderkrankenschwester 2013).

Forschern der Queensland University of Technology in Brisbane ist es gelungen, eine Oberfläche zu entwickeln, auf der multiresistente Keime nicht wachsen können und die in Krankenhäusern eingesetzt wird. Die Struktur dieser Oberfläche ist der Oberfläche von Libellenflügeln nachempfunden (Jaggessar et al. 2017).

Die aktuell vielversprechendste Methode, die Bakteriophagen-Therapie, nutzt Bakteriophagen, Bakterien befallende Viren, um bakterielle Infektionen zu bekämpfen. Im Vergleich zu den chemischen Antibiotika besitzen Phagen einige Vorteile. Zum einen sind sie in der Lage, die menschliche Blut-Hirn-Schranke zu durchdringen. Zum anderen gibt es in der Bakteriophagen-Therapie keine negativen Nebenwirkungen, da Phagen als auf Prokaryoten spezialisierte Viren menschliche Zellen nicht befallen können. Dass diese Therapie wirkt, zeigt unter anderem ein Fall aus England, bei dem eine Infektion mit *Mycobacterium abscessus* bei einer Mukoviszidosepatientin erfolgreich mit Phagen therapiert wurde. Die Ärzte gaben an, dass die Überlebenschance der Patientin auf unter 1 % geschätzt wurde (Gallagher 2019).

Die Therapieform geht auf Forschung aus dem zwanzigsten Jahrhundert aus der damaligen UdSSR zurück (Myelnikov 2018). Aus diesem Grund ist sie im östlichen Europa heute populärer als im Westen. Führend in der Anwendung der Bakteriophagen-Therapie ist Georgien. Durch die steigenden Antibiotikaresistenzen erlebt die Therapie eine Renaissance in der biomedizinischen Forschung (Bragg et al. 2018; Domingo-Calap und Delgado-MartÍnez 2018; Gordillo Altamirano und Barr 2019). Auch der Bayerische Landtag diskutierte im Antrag „Todesfälle durch multiresistente Keime vermeiden V“ den möglichen Einsatz (Seidenath et al. 2019b).

Andere Studien zeigten, dass bereits eine Therapie mit gezielterem Antibiotikaeinsatz die Situation verbessern kann. So spart ein gezielterer Einsatz von Antibiotika bei Streptokokken-Infektionen nicht nur fast die Hälfte des Wirkstoffes ein, sondern erzielt auch dieselbe Wirkung wie die ursprünglichen Therapien (Tetsch 2020). Die Wirkung der Homöopathie ist dagegen bis heute nicht über den Placebo-Effekt hinaus bewiesen. Trotzdem boomt das Geschäft mit alternativmedizinischen Methoden, von denen die Homöopathie die beliebteste und auch offiziell von vielen anerkannt ist. Der stattgegebene Antrag im Bayerischen Landtag ist dabei nur ein Beispiel. Ein anderes ist die Übernahme der Kosten von homöopathischen Behandlungen durch viele Krankenkassen. Der bekannteste Hersteller von Homöopathika ist die in Nussbaum angesiedelte Firma Hevert-Arzneimittel. Diese erzielte im Jahr 2018 einen Umsatz von 29,7 Mio. €. Nachdem Hevert im Jahr 2019 Unterlassungserklärungen von mehreren Homöopathiekritikern, unter anderem von der Ärztin Natalie Grams, verlangte, rückte das kleine mittelständische Unternehmen in den Fokus der Öffentlichkeit. Viele Personen, unter anderem SPD-Politiker wie der Epidemiologe Karl Lauterbach, kritisierten das Vorgehen des Unternehmens. Das Geschäft wurde von dieser Kritik allerdings nicht beeinflusst (Hofmann 2019).

## Ethische Fragen

Durch den Beschluss, Homöopathika als Ersatz für Antibiotika zu erforschen, grätscht der Bayerische Landtag in gewisser Weise in die aktuelle Phase der biomedizinischen Innovation. Dieser Beschluss wirft mehrere ethische Probleme auf.

1. Die Politiker berufen sich in ihrem Antrag auf eine bestimmte Studie aus dem Jahr 2005. In der Studie *Adjunctive Homeopathic Treatment in Patients With Severe Sepsis: A Randomized, Double-Blind, Placebo-Controlled Trial in an Intensive Care Unit*, die vom Wiener Homöopathen Michael Frass durchgeführt und im Journal *Homeopathy* veröffentlicht wurde, wird die Wirkung von homöopathischen Mitteln auf Patienten mit schwerer Sepsis beschrieben (Frass et al. 2011; Ernst 2011). Die Antragsteller um Bernhard Seidenath schreiben in Drucksache 18/3320: „Wissenschaftliche Studien, besonders im Bereich HNO-Erkrankungen, konnten aufzeigen, dass durch den Einsatz klassischer Homöopathie sowohl der Einsatz von Antibiotika vermieden als auch eine Verbesserung der individuellen Infektabwehr erreicht werden konnte. Weiterhin wies eine Studie zur Mortalität von Patienten mit einer schweren Sepsis darauf hin, dass eine homöopathische Behandlung eine nützliche zusätzliche Behandlungsmethode bei schwer septischen Patienten darstellen kann“ (Seidenath et al. 2019a). Diese Behauptung ist grob falsch. Die Sepsisstudie, auf die im Antrag hingewiesen wird, ist die bereits genannte Studie aus dem Jahr 2005 von Frass et al. Diese wurde aufgrund verschiedener Fehler allerdings stark kritisiert. So wurden sämtliche Patienten regulär mit Antibiotika behandelt. Lediglich ein Teil wurde zusätzlich mit Homöopathika behandelt. Die Nutzung von Antibiotika verfälschte die Ergebnisse deutlich, da die gesundheitliche Verbesserung der Patienten nicht auf die homöopathische Nutzung zurückgeführt werden kann. Des Weiteren beschreibt die Studie lediglich die Wirkung auf Patienten mit schwerer Sepsis. In der Studie wurden allerdings auch Patienten mit anderen Beschwerden behandelt, unter anderem mit *respiratory insufficiency*. Aufgrund dieser Fehler kann nicht davon gesprochen werden, dass Homöopathie die Gesundheit der Patienten verbessert (Aust 2014). Die Studie wird regelmäßig kritisiert. Michael Frass wiederum verteidigt seine Studie (Aust 2016).
2. Das Internet ist heute eine der Hauptquellen für Informationen aller Art. Allerdings schwanken diese in ihrer Qualität und Seriosität deutlich. So ist die wissenschaftliche Literatur für Laien nicht immer leicht zugänglich. Diese weichen folglich auf alternative Quellen aus. Dies wird unter anderem dadurch verdeutlicht, dass die am meisten genutzten Suchmaschinen neben Google das Videoportal YouTube und die Social-Media-Plattform Facebook sind (PromoMasters 2020). Beide Seiten sind deutlich leichter zugänglich als wissenschaftliche Suchmaschinen wie zum Beispiel PubMed. Auf Facebook organisieren sich viele Alternativmediziner und Homöopathiker in geschlossenen Gruppen, um dort medizinische Probleme zu diskutieren und um sich auszutauschen. Da Kritiker aus diesen Gruppen oft entfernt werden, entstehen gefährliche Informationsblasen. Auch auf der Videoplattform YouTube kursieren viele Videos von Alternativmedizinern und medizinischen Laien. Im Grunde kann jeder einen eigenen YouTube-Kanal eröffnen und Videos hochladen. Dazu kommt, dass einige Hersteller gezielt mit Falschinformationen werben. So verwischen Hersteller von homöopathischen Präparaten innerhalb ihres Marketings wissentlich die Grenze zwischen Homöopathie und Naturmedizin (Sarma 2010). Um in der Lage zu sein, diese Falschinformationen von richtigen Fakten, die auch auf YouTube gefunden werden können, unterscheiden zu können, ist eine umfassende Medienbildung und Medienkompetenz erforderlich (Barberi 2017). Wie wichtig eine Bildung in diesem Bereich ist, zeigt unter anderem eine Allensbach-Umfrage, nach der lediglich 17 % der Bevölkerung wussten, was Homöopathie wirklich ist. 74 % ordneten die Homöopathie innerhalb der Naturheilkunde ein. Da innerhalb der Homöopathie auch nicht-pflanzliche Mittel eingesetzt werden, ist diese Bezeichnung nicht zutreffend (Sarma 2010). Seit kurzem gehen sowohl YouTube als auch Facebook gegen Fake News vor. So rief Facebook erst vor kurzem seine User auf, falsche Informationen an die Firma weiterzuleiten (Scott 2020). Auch YouTube geht vermehrt gegen Falschinformationen vor, wobei die amerikanischen Firmenregeln, zu denen auch die *Freedom of Speech* gehört, mit der *Meinungsfreiheit* der Europäischen Union aneinandergeraten (Stolton 2019). Als einem Unternehmen aus den Vereinigten Staaten spielt die *Freedom of Speech* bei YouTube eine wichtige Rolle. Im Gegensatz zur europäischen *Meinungsfreiheit* deckt die *Freedom of Speech* auch Meinungen, die beleidigend oder verleumderisch sind. Die *Meinungsfreiheit* in Deutschland ist ein Teil des Grundgesetzes, wird aber durch andere Grundrechte soweit eingeschränkt und reguliert, dass Beleidigungen und andere Äußerungen nicht geschützt werden. Wissenschaftliche und medizinische Falschinformationen, die mitunter sehr gefährlich sein können, werden durch die *Freedom of Speech* geschützt, durch die *Meinungsfreiheit* jedoch nicht (Bambauer 2017).
  Wie schnell sich alternative Heilungsmethoden heutzutage verbreiten, zeigt die aktuelle COVID-19-Pandemie. Im Internet kursieren viele verschiedene Behandlungsmethoden, von denen einige lediglich belustigend, andere aber durchaus gefährlich und lebensbedrohlich sind. Während in der Vergangenheit die Nutzer und Vertreter dieser Methoden von Wissenschaftlern lediglich belächelt wurden, gibt es mittlerweile eine positive Entwicklung in der wissenschaftlichen Gemeinschaft. So rief ein vor kurzem erschienener Artikel im *Nature* auf, diese so genannte *Pseudoscience* nicht länger zu tolerieren (Caulfield 2020). Dabei spielen neben Wissenschaftlern und Ärzten auch Politiker eine essentielle Rolle. So nahm zum Beispiel die Anzahl der Giftnotrufe in den Vereinigten Staaten deutlich zu, nachdem Präsident Donald J. Trump in einer Pressekonferenz fragte, ob eine Injektion mit Desinfektionsmitteln als mögliche Behandlung in Betracht käme (Noor 2020). Der stattgegebene Antrag des Bayerischen Landtages geht in eine ähnliche Richtung. Denn entgegen der allgemeinen Annahme, dass es sich bei Homöopathika um harmlose Zuckerkügelchen handele, gibt es auch hier Gefahren bei der Einnahme. So kam es in den USA zu zehn Todesfällen bei Kleinkindern, nachdem diese sogenannte *Teething Tablets* einnahmen. Dieses homöopathische Mittel soll die Schmerzen von zahnenden Kleinkindern reduzieren. Als Wirkstoff dient Belladonna, ein Giftcocktail der aus der Schwarzen Tollkirsche *Atropa belladonna* gewonnen wird (ZEIT ONLINE 2017). Mit einem LD50-Wert von 75 mg/kg (Wirkstoff Atropin in der Maus) gehört *A*. *belladonna* zu den giftigsten Pflanzen der Welt (DrugBank 2017). Zu den Symptomen gehören unter anderem Ataxie, Desorientierung, Verlust des Kurzzeitgedächtnisses, Verwirrtheit, Halluzinationen, Psychosen, Atemstillstand und kardiovaskulärer Kollaps (Berdai et al. 2012). Die Vergiftungen in den USA sind auf eine fehlerhafte Verdünnung zurückführbar (ZEIT ONLINE 2017).
3. Ein weiterer Kritikpunkt ist die Frage der Notwendigkeit einer weiteren Wirkungsstudie. Seit ihrem Aufkommen im 18. Jahrhundert wurden unzählige Studien, die sich mit der Homöopathie und ihrem Wirken auseinandergesetzt haben, durchgeführt. Die Onlinedatenbank PubMed listet aktuell 5934 Ergebnisse bei der Suche nach *Homeopathy*, 449 bei der Suche nach *Homeopathy efficacy* und 158 Ergebnisse bei der Suche nach *Homeopathy metaanalysis* (Stand Mai 2020).
  Die Arbeit *Evolution of Homeopathy*, die Anfang 2019 in *Complementary Therapies in Clinical Practice* veröffentlicht wurde, zählt 4183 Artikel. 72,75 % von diesen sind Originalartikel. Industrienationen dominieren dabei die Veröffentlichungen deutlich. Die meisten Artikel wurden im Vereinigten Königreich veröffentlicht (950 Artikel), gefolgt von den USA (636 Artikel), Deutschland (590 Artikel), Indien (277 Artikel) und Brasilien (246 Artikel). Allerdings führt die Schweiz im Bereich der Produktivität mit einem Wert von 20,41, gefolgt vom Vereinigten Königreich (14,35), Norwegen (11,31) und Israel (8,41). 4,88 % der Veröffentlichungen stammt von der britischen University of Exeter. Das führende Journal im Bereich ist *Homeopathy*, in dem 24 % aller homöopathischen Artikel veröffentlicht werden (Şenel 2019).
  Innerhalb der Veterinärwissenschaft, in der Homöopathie ebenfalls eine Rolle spielt, wurden 52 Trials in 48 Veröffentlichungen gezählt (Stand 2016). Von diesen erzielten 26 einen signifikant besseren Effekt, während 22 keinen besseren Effekt erzielten. Alle diese Studien wurden nicht wiederholt, weshalb ebenfalls nicht von einer Wirkung über den Placeboeffekt gesprochen werden kann (Doehring und Sundrum 2016).
  Studien, die einen positiven Effekt der Homöopathie aufzeigen, gibt es vereinzelt. Allerdings konnten diese Ergebnisse bis heute nicht wiederholt und somit auch nicht gefestigt werden. Eine Wirkung über den Placeboeffekt ist bis heute nicht erbracht (Linde 1999).
  Viele Studien, die auf den ersten Blick einen positiven Effekt der Homöopathie nachweisen, weisen bei näherer Betrachtung Fehler auf, die das Ergebnis der Studie teilweise deutlich verfälschen. Dazu gehört auch die weiter oben bereits beschriebene Sepsisstudie aus dem Jahr 2005. So kommt es allgemein oft zu Fehlern innerhalb des Prozesses der Randomisierung. Andere Fehler entstehen zum Beispiel durch die hohe Abbruchquote der Probanden innerhalb der Studien. Gerade bei der Homöopathie spielt die Potenzierung eine wichtige Rolle. Dadurch kommt es aber auch innerhalb der Forschung zu weiteren Fehlerquellen, da unterschiedliche Potenzierungen innerhalb der homöopathischen Lehre unterschiedlich wirken und somit auch unterschiedliche Ergebnisse erzielen sollten. Unterschiedliche Potenzierungen sollten deswegen nicht miteinander verglichen werden (Linde 1999).
  Darüber hinaus gibt es noch weitere Probleme mit der Publizierung diverser Ergebnisse. Wie bereits beschreiben ist das führende Journal *Homeopathy*. Dieses veröffentlicht Artikel und Ergebnisse aus der homöopathischen Forschung. Kritische Artikel wiederum finden keinen Platz im Journal, weshalb es hier, wie auch bereits in den Gruppen auf Social-Media-Plattformen, zu einem Informationsungleichgewicht kommt. Gute wissenschaftliche Journals sollten aber gerade auch kritische Stimmen zulassen, da Kritik und Diskussion die Grundlagen guter Forschung ausmachen. Seit 2018 gehört das Journal zum Thieme Verlag.
  Neben diesen offiziellen Veröffentlichungen gibt es auch noch das Problem mit nicht-offiziellen und unseriösen Veröffentlichungen. Dr. Linde beschreibt dies in seiner Arbeit *Gibt es gesicherte Therapien in der Homöopathie?*, die 1999 in *Der Internist* veröffentlicht wurde, anhand einer französischen Studie über den Einsatz von Nosoden bei Influenza. Casanova und Gerard konnten in einer Studie einen positiven Effekt von Nosoden aufzeigen. Diese Arbeit litt allerdings sowohl an ihrer schlechten Methodik als auch an ihrer sehr unseriösen Publizierung. Die von Linde beschriebene Studie ist zur Verdeutlichung in den Referenzen aufgeführt, siehe Casanova (1984) sowie Casanova und Gerard (1992), Linde (1999).
4. Forschern sollte man nicht vorschreiben, woran sie forschen. Diese Freiheit ist essentiell in der Wissenschaft. Ethik und Philosophie bestimmen die nötigen Grenzen und verhindern, dass Forscher menschliche Grenzen überschreiten. Allerdings sollte auch der gesellschaftliche Nutzen eine Rolle spielen, gerade wenn die Forschung in staatlichen Instituten stattfindet. Da die Studie, die vom Bayerischen Landtag beschlossen wurde, durch Steuergelder bezahlt wird, muss der Nutzen diskutiert werden. Bei der Menge an Studien, Veröffentlichungen und anderen Informationen ist es fragwürdig, ob diese Studie neue Erkenntnisse liefert und ob das Geld der Steuerzahler nicht besser investiert werden kann, zum Beispiel durch die Finanzierung einer Studie über den Nutzen von Bakteriophagen. Im Antrag „Todesfälle durch multiresistente Keime vermeiden V“ (Drucksache 18/3321) wird die Möglichkeit einer solchen Studie NICHT benannt. Anders im Homöopathieantrag „Todesfälle durch multiresistente Keime vermeiden IV“ (Drucksache 18/3320). Es ist auch fraglich, wie die geplante Studie aussehen soll. Um verwertbare Ergebnisse zu erzielen, müssten an resistenten Keimen erkrankte Patienten mit Homöopathika und mit anderen Placebos als Kontrolle behandelt werden. Antibiotika dürfen dabei nicht eingesetzt werden, da ihr Nutzen das Ergebnis verfälschen würde. Dies passierte bei der Sepsisstudie aus dem Jahr 2005 (Frass et al. 2011; Aust 2014). Ethisch ist dieser Vorgang allerdings nicht tragbar, da Patienten eine wichtige Behandlung vorenthalten wird.

## Fazit

Multi-resistente Keime gehören zu den gefährlichsten und somit aktuell auch zu den wichtigsten Herausforderungen der modernen Medizin. Neue Ansätze sind wichtig, um diese Gefahr zu minimieren und letztendlich Patientenleben zu retten. In diesem Artikel haben wir einige Beispiele für Alternativen genannt. Als am meisten erfolgversprechend ist wahrscheinlich die Bakteriophagentherapie. Allerdings kommuniziert der Bayerische Landtag mit seiner Entscheidung, Homöopathie als Alternative zu erforschen, einen falschen, ethisch problematischen Weg. Homöopathie ist eine umstrittene alternativmedizinische Methode. Seit der Entwicklung der homöopathischen Lehre im 18. Jahrhundert fehlt ein wissenschaftlicher Beweis für die Wirksamkeit. Da diese Lehre naturwissenschaftlichen Gesetzen widerspricht, ist es sehr wahrscheinlich, dass dieser Nachweis auch in Zukunft nicht erbracht werden kann. Trotzdem wird die Homöopathie und werden auch andere alternativmedizinische Methoden immer beliebter. Durch seinen Vorstoß fördert der Bayerische Landtag diese Entwicklung. Doch dadurch entstehen einige Probleme. So greifen Patienten auch bei schweren Erkrankungen zuerst zu Globuli und erst später zu wirkenden Mitteln. Allerdings ist es dann meistens bereits zu spät. Dieser, aber auch andere Gründe sorgen dafür, dass auch heutzutage die Homöopathie immer noch heiß diskutiert wird.
